# Description of a New and Economical Fracture Box

**Published:** 1831-08

**Authors:** J. Russell

**Affiliations:** Surgeon, &c.


Plate(s) missing

THE LONDON
### Medical and Physical Journal.

390, VOL. LXVII.] AUGUST 1831. [62, *New Series*.

"For many fortunate discoveries in medicine, and for the detection of numerous errors, the world is indebted to the rapid circulation of Monthly Journals; and there never existed any work to which the Faculty, in Europe and America, were under deeper obligations than to the 'Medical and Physical Journal of London,' now forming a long but invaluable series."—RUSH.

### ORIGINAL PAPERS, AND CASES,
### OBTAINED FROM PUBLIC INSTITUTIONS AND OTHER AUTHENTIC SOURCES.

### NEW FRACTURE BOX.

### *Description of a New and Economical Fracture Box.*
By J. RUSSELL, Surgeon, &c.

[WITH A PLATE.]

HAVING lately had occasion to treat a severe case of compound fracture of the leg, in which the ordinary splints and apparatus were insufficient to effect the various objects required, I was induced to construct a fracture box that perfectly answered the purpose, and which, from its great simplicity and universal adaptation, appears worthy of more general notice. It has long been a desideratum with surgeons, and more particularly with country general practitioners, to possess an apparatus that would supersede the necessity of having a great number of splints, and which could be adapted to every variety of fractured leg that might occur, and at the same time be so simple in construction and application, and of such moderate expense, as would render it a part of the ordinary apparatus in the hands of every practitioner. The fracture box I desire to introduce to the notice of the profession, appears better calculated to effect these objects than any with which I am acquainted; for although it may not effect more than various others already known to the profession, it is less complex and expensive, and may, therefore, most likely, be generally adopted.

Every surgeon, who has had the treatment of fractures of one or both bones of the leg, must have experienced the inconvenience attending the use of the ordinary splints in reducing the fracture: under their use, the only available means of keeping the bones in their situation is by position, or pressure at the sides and round the whole circumference of the limb. But as, frequently, the limb is severely bruised, and subsequently swollen and inflamed, pressure upon it is impossible, the surgeon has only the alternative of trusting to position, and leaving the bones imperfectly restored to their natural situation, or of keeping up continual irritation by the pressure on the swollen limb. Those results can only be prevented by fixing the foot and ankle at one point, and the heads of the bones at the knee firm and steady at the other: when these objects are effected, the slightest support will be sufficient to keep the fractured ends of the bones in apposition; and leeches, lotions, &c. may be applied to the inflamed parts without inconvenience. In compound fracture, the wound may require frequent or daily dressing, and it is desirable that this object be effected without an risk or disturbance to the bones: in the case to which I have alluded, suppuration had taken place round the fractured ends of the bones; frequent poultices were required, and were constantly applied by the ordinary attendant, without the slightest inconvenience or risk of displacement.

The fracture box consists of a flat deal or mahogany board, (No. 1,) five inches and three quarters wide, and thirty-three inches long, jointed with two hinges at eleven inches from the extremity; a perforated brass or iron plate (A), about nine inches long, is let in at the extremity of the longer portion, and on this the footboard slides. The footboard is a piece of wood, (Nos. 2 and 3,) five inches and three quarters wide, and twelve inches high, having a metal band, (B,) one inch and a half wide, passing underneath, allowing space for the flat board to go accurately between: the footboard thus slides backwards and forwards; it has a metallic plate projecting from the back, (C,) through which a screw passes into the perforated plate, and two or three turns of the screw make it firm and secure from motion at any required length. Six openings are to be made in the board, as at D, for tapes to pass through. The other parts consist of three sliders, (No, 4,) which are formed each of three pieces of wood, one at each side (E, E,) six inches and a half high on the outside, and four and one eighth inches wide, and one passing underneath, (F,) to which the others are dovetailed. The side pieces project a little over the board, allowing an intermediate space of five inches, and the sliders thus pass freely backwards and forwards, but cannot be removed except at the ends. Additional strength is given by two metallic bands (G, G, No. 6,) passing outside, to prevent them yielding to any degree of force. In the centre of that portion of each side of the sliders opposite to the edge of the board, (H, No. 6,) a screw is fitted in a plate; by one or two turns of which the sliders are firmly secured at any particular part, and, as the screws may be removed to either side, will not interfere with the opposite leg: a piece of stout linen or calico, shaped as at No. 5, with tapes and buckles attached, to pass through the openings, binds the foot to the footboard: a strap, with buckle, to be placed across the thigh above the knee, completes the apparatus.

When this fracture box is used, a pad is to be made the whole length of the board, and adapted to the hollow between the ankle and heel, or this part may be supported by an additional pad. Another pad is fixed between the foot and the footboard; and pads are to be made for each side of the sliders, a little larger than the slides. The last named are to be made of a thickness corresponding to the size of the limb to which they are to be applied. All these pads would be best made of straw, with one outer layer of tow, and covered with calico: the whole may be made by any person in the course of twenty minutes, and, if ready prepared, they may be adapted to the particular limb by additional padding of tow between the sliders and pads: it will, however, be better to make them according to each particular case, preserving one or two pads as patterns.

When about to be applied to a fractured limb, it will be necessary to adjust the footboard to the exact length of the leg, by fitting it to the sound limb; the joint is to fit exactly under the knee, making allowance for the pad. The exact adjustment of length will be a point of some consequence: this, however, will be materially assisted by regulating the thickness of the pad for the foot. When once thus adjusted, the application is easy: the foot is to be placed on the pad, and the foot bandage placed on the instep, the long piece passed round the heel, and the tape put through the loop; the tapes are then to be passed through the openings in the footboard, and buckled. The foot is thus tied firmly down to the pad and footboard, with the ease and security of a laced stocking.

If the limb is intended to be placed either on the inclined plane or on the side, one slider is to be placed directly above the knee, and the other immediately below; the pads are then to be fixed at the sides: they should press somewhat firmly, and be secured by a piece of tape at the back, which enables each to be secured to the opposite pad. The third slider is to be placed opposite the fracture, and the pads, carefully fitted in the slider, are to be placed in like manner, when it will be found that the bones will be perfectly firm and secure, and will enable the position of the limb and box to be changed, without risk of displacement: one or two turns of each screw will fix the sliders firmly in their position. When necessary to examine the limb, the two side pads are to be moved away, and the sliders slipped upwards or downwards along the board.

If necessary to make any application to the limb, the longer pad may be covered with oil silk; and leeches, &c. may then be applied, with perfect convenience. If advisable to apply pressure to the bones while these applications are being used, one side may be supported by a pad, while applications are made to the other parts. Where a projecting portion of bone requires pressure, the pad may be so adjusted as to press on a small point, without affecting the surrounding parts.

When it is considered desirable to place the limb in a straight position, which is most likely chosen in the early stage of severe fracture, one slider is to be brought over the joint, and screwed, when the whole becomes at once a straight firm splint, (the sliders being grooved to pass over the hinges.) One or both the other sliders may then be used opposite the fracture; as, in particular cases, both may be requisite along the course of the fractured bones.

From this account of the construction and application of the fracture box, it will be seen that it is adapted to all sizes, to every variety of position, and to any description of fracture of the leg: it will admit of the examination of the limb as often as necessary, without the slightest disturbance, as the pads and slides are instantaneously removed and reapplied. Applications may be used by the ordinary attendants without any risk, the firm manner in which the foot and knee are fixed rendering it almost impossible to displace the bones. Another advantage of this fracture box is, that it is capable of being made a very complete thigh splint, by adapting an additional board to the upper part; thus fulfilling the object of having one apparatus for every kind of fracture of the lower extremity. For this purpose a second plate is to be fixed the whole length of the shorter portion of the board, with perforations one inch asunder; and the second board made three fourths of an inch narrower than the former in half its length: on the under surface, bolts, one inch apart, with screws and nuts adapted, are to be fixed. When used as a thigh splint, this board is to be laid over the other, the bolts passed through at the required length, and the nuts firmly screwed. Two small side splints, eight inches by three, with two straps and buckles, will complete all that is necessary for the thigh.

When the apparatus is thus constructed, it will admit of three positions: first, as a straight splint under the thigh; second, as a straight one on the outside, by removing the sliders; and third, as an inclined plane of any required length, and supported in the usual manner of the ordinary fracture boxes.

The description I have given of the apparatus, will probably be a sufficient guide to any instrument maker for its proper construction. It has been manufactured for me, in a neat manner, and at a very moderate expense, by Mr. J. Deane, 1, Sidney street, near the London Hospital.

*Description of the Plate.*

No. 1. Flat board.
(*a*) Metal plate, with perforations for screws.
(*p*) Dotted line for an additional plate, when constructed as a thigh splint.
(*r*) Joint, with two strong hinges underneath.
No. 2. Foot board.
(*d, d*) Perforations for tapes.
(*b*) Metal plate, allowing space for the long board to pass between.
No. 3. Side view of foot board.
(*c, c*) Projecting metal plates.
No. 4. (*e, e*) Upright sliders, sides grooved at the lower point.
(*f*) Bottom grooved to pass over the hinges.
No. 5. Bandage for tying the foot to the footboard.
(*i, i, i*) Tapes with buckles.
(*k, k*) Ditto, to attach to the buckles.
(*m*) A piece to pass round the heel, and through the loop *l*.
(*l*) Loop.
No. 6. Fracture box, with two sliders in the straight position.
(*g, g*) Metal bands.
(*h*) Screw for fixing the sliders.
(*n*) Joint rendered a straight splint, by one slider passed over.
No. 7. Fracture box, with three sliders, in an inclined position.
No. 8. Additional board, when constructed as a thigh splint.
(*o, o*) Stout screw bolts, to pass through perforated plate, *p*, fig. 1.
(*q*) Opening for thigh strap.

